# Incidence trends in prostate cancer among men in the United States from 2000 to 2020 by race and ethnicity, age and tumor stage

**DOI:** 10.3389/fonc.2023.1292577

**Published:** 2023-11-30

**Authors:** Xianglin L. Du, Daoqi Gao, Zhuoyun Li

**Affiliations:** Department of Epidemiology, Human Genetics and Environmental Sciences, School of Public Health, The University of Texas Health Science Center at Houston, Houston, TX, United States

**Keywords:** prostate cancer, cancer incidence, incidence trend, racial disparities, SEER areas

## Abstract

**Purpose:**

To explore whether prostate cancer incidence trends from 2000 to 2020 in the United States differed by race and ethnicity, age and tumor stage; to explore racial differences in prostate cancer incidence change due to the impact of COVID-19 pandemic lockdown in 2020; and to determine if there is any high-risk population that can be targeted for prevention.

**Methods:**

We identified 1,098,349 men who were diagnosed with incident prostate cancer at age ≥20 in 2000-2020 in 17 registries of the Surveillance, Epidemiology, and End Results (SEER) program in the United States; of whom, 778,437 were non-Hispanic whites, 155,111 non-Hispanic blacks, 4,200 American Indians and Alaska Natives (AIAN), 55,267 non-Hispanic Asians/Pacific Islanders, and 105,334 Hispanics.

**Results:**

Age-adjusted incidence rate of prostate cancer was the highest in blacks (302.6 cases per 100,000 men), followed by whites (186.6), Hispanics (153.2), AIAN (108.5), and Asians (104.9). Age-adjusted prostate cancer incidence rates dramatically decreased from 2000 to 2013 for all ethnic men. However, age-adjusted prostate cancer incidence rates increased from 2014 to 2020, in which the increasing incidence trend looked sharper in blacks and whites, flatter in Asians, and leveled in AIAN and Hispanics. Among men with local or regional stages across all years, prostate cancer incidence rate was significantly higher in blacks, but significantly lower in Hispanics, AIAN, and Asians as compared to whites. Among men in 2007-2013, the risk of distant stage prostate cancer was statistically significantly elevated in blacks (rate-ratio: 2.22, 95% CI: 2.06-2.38) and Hispanics (1.16, 1.06-1.25), not significantly different in AIAN (1.30, 0.92-1.76), but still significantly lower in Asians (0.73, 0.66-0.82) as compared to whites. There was a drop of prostate cancer incidence from 2019 to 2020 likely due to the impact of COVID-19 pandemic lockdown on the access to medical care in 2020. Overall prostate cancer incidence rate decreased by 40.4 cases per 100,000 population from 277.4 in 2019 to 237.0 in 2020 for blacks, 20.9 from 164.2 to 143.3 for whites, 16.8 from 124.8 to 108.0 for Hispanics, 14.9 from 101.7 to 86.8 for AIAN, and 12.6 from 88.4 to 75.8 for Asians.

**Conclusion:**

The decreasing trend of prostate cancer incidence from 2000 to 2013 was statistically significant for all ethnic men. There was an increasing prostate cancer incidence from 2014 to 2020. Age-adjusted incidence rate of prostate cancer was the highest in blacks, followed by whites, Hispanics, AIAN, and Asians, regardless of age groups, tumor stages, and time periods. There will also be a need to monitor and investigate the prostate cancer incidence trend during and after COVID-19 pandemic season.

## Introduction

Prostate cancer incidence in men has been increasing globally from 1990 to 2017 in 21 regions, including 195 countries and territories across Asia, Africa, Europe, Middle East, and North America (1–12). For example, the age-standardized incidence rate of prostate cancer increased from 30.5 cases per 100,000 population in 1990 to 37.9 cases per 100,000 population in 2017 (2). The highest incidence was observed in high-income countries (2, 3). The crude prostate cancer incidence increased from 115 to 137 cases per 100 000 males in Canada from 1992 to 2010 (3). However, in the United States, the incidence of prostate cancer increased sharply from 1975 to 1991 with the peak in the early 1990s due to the widespread use of prostate-specific antigen (PSA) screening through which many men with asymptomatic prostate cancer were detected (1). The incidence of prostate cancer then decreased tremendously from 1991 to 2013, but then increased again by 3% per year from 2014 to 2019, which was translated to 99,000 more prostate cancer cases than would have occurred if the incidence rates remained stable (1). About half of these additional prostate cancer cases that occurred were found at an advanced tumor stage (1). This phenomenon happened likely because the United States Preventive Services Task Force (USPSTF) recommended against screening for men aged 75 years and older in 2008 and for all men in 2012 (13–17). Good news is that prostate cancer mortality has continued to decline (1). Although several studies examined racial disparities in prostate cancer incidence, tumor stage and mortality in recent years (18–22), they did not examine the prostate cancer incidence trend over time by race and ethnicity. Therefore, this study aimed to make some unique contributions to the existing literature: 1) to utilize the latest cancer registry data released in April 2023 to study the prostate cancer incidence trends from 2000 to 2020 in the United States by race and ethnicity, age and stage, including racial disparities in unknown tumor stage; 2) to explore racial differences in prostate cancer incidence change due to the impact of COVID-19 pandemic lockdown in 2020; and 3) to determine if there is any high-risk population that can be targeted for screening, prevention and intervention in order to decrease the prostate cancer occurrence and reduce the disparities.

## Methods

### Data sources

This study utilized the National Cancer Institute’s Surveillance, Epidemiology, and End Results (SEER) Public Use Datasets that were made available for researchers in April 2023. Both prostate cancer cases (numerator) and population (denominator) from SEER areas in the United States from 2000 to 2020 are available for cancer incidence calculation (23). The SEER program supports 17 population-based tumor registries in 8 areas (San Francisco/Oakland, San Jose-Monterey, Los Angeles, Greater California, Seattle, Atlanta, Rural Georgia, and Greater Georgia) and 9 states (Alaska, Connecticut, Iowa, New Mexico, Utah, Hawaii, Louisiana, Kentucky, and New Jersey), accounting for 28% of the U.S. population. The SEER registries ascertain all newly diagnosed (incident) cancer cases from multiple reporting sources. The estimated completeness of cancer case reporting in SEER areas was 97.7% (23). This study was considered exempt for Institutional Review Board (IRB) review because it did not involve any patient contact, only had the analysis of de-identified SEER Public Use Data, and had no any health risk to study subjects.

### Study population

Our study identified 1,098,349 men who were diagnosed with incident prostate cancer (International Classification of Diseases-Oncology, ICD-O-3 code C61.9) at age 20 or older between 2000 and 2020 in 17 SEER registries. The denominator of population data included all 608,686,103 men aged 20 or older in the same SEER areas that were provided in the SEER*Stat package (23). Of the 1,098,349 men with prostate cancer, 778,437 were non-Hispanic whites, 155,111 were non-Hispanic blacks, 4,200 were American Indians and Alaska Natives, 55,267 were non-Hispanic Asians/Pacific Islanders, and 105,334 were Hispanic men.

### Study variables

Race and ethnicity variable was classified into non-Hispanic white (white), non-Hispanic black (black), American Indians and Alaska Natives (AIAN), non-Hispanic Asians/Pacific Islanders (Asian), and Hispanic men. Patients with unknown or missing information on race and ethnicity were included for the incidence calculation for overall population and some sup-total stratified groups, but were not presented as a separate race and ethnicity group because the SEER*Stat software did not provide the incidence information for this group. Patient age was divided into 3 broad groups as young (20-44 years), middle-age (45-54), and older age (≥55) in order to avoid small number of cases in stratified analyses by race and ethnicity, tumor stage, and year of diagnosis. The other covariates included year of diagnosis (2000 to 2020) and SEER areas by state where 17 SEER registries were located (23). Tumor factor included tumor stage (local, regional, distant stage, or unknown/missing).

### Statistical analysis

We utilized the SEER*Stat software (version 8.4.0.1) that was provided by the National Cancer Institute together with the SEER Data for analyses in cancer incidence rates and trends. Incidence of prostate cancer in men was defined as a ratio of the number of men with a new prostate cancer over the number of total male population at risk in the same SEER areas by year, which was presented as the number of cases per 100,000 persons. Because age is a significant risk factor for cancer incidence, the incidence rates of prostate cancer in all comparison groups by race and ethnicity or time periods that may consist of different age compositions should be standardized by age. Therefore, in this study the incidence rates were adjusted to the year 2000 U.S. population by age. The age-adjusted incidence rates, 95% confidence intervals for incidence rates, incidence rate ratios (IRR), 95% confidence intervals for rate ratios, and annual percentage change (APC) of incidence rates were calculated from the SEER*Stat software. A p value <0.05 was considered statistically significant.

## Results

**Table 1** presents the number of total male population, number of incident prostate cancer cases, unadjusted and age-adjusted incidence rates by race and ethnicity in men aged 20 or older in all 17 SEER areas in 2000-2020. The overall age-adjusted incidence rate of prostate cancer was the highest in blacks (302.6 cases per 100,000 men), followed by whites (186.6), Hispanics (153.2), AIAN (108.5), and Asians (104.9). The incidence rate ratio was statistically significantly higher in blacks (rate ratio: 1.62, 95% CI: 1.61-1.63), but was significantly lower in AIAN (0.58, 0.56-0.60), Asians (0.56, 0.56-0.57), and Hispanics (0.82, 0.82-0.83) as compared to whites.

**Table 1:** Number of population, number of incident prostate cancer cases, and prostate cancer incidence in men by race and ethnicity in all SEER areas, 2000-2020.

Race and Ethnicity	Number of population	Number of prostate cancer cases	Unadjusted Incidence rates (N of cases per 100,000) (95% CI)	Age-adjusted* incidence rates (N of cases per 100,000) (95% CI)	Rate ratio (between age-adjusted incidence rates) (95% CI)
**Non-Hispanic (NH) white**	354638804	778437	219.5 (219.0-220.0)	186.6 (186.1-187.0)	1.00 (REF)
**NH black**	61901361	155111	250.6 (249.3-251.8)	302.6 (301.0-304.2)	1.62 (1.61-1.63)
**NH American Indians/ Alaska Natives**	4873194	4200	86.2 (83.6-88.8)	108.5 (105.0-112.0)	0.58 (0.56-0.60)
**NH Asians/ Pacific Islanders**	60842746	55267	90.8 (90.1-91.6)	104.9 (104.0-105.8)	0.56 (0.56-0.57)
**Hispanics**	126429998	105334	83.3 (82.8-83.8)	153.2 (152.2-154.2)	0.82 (0.82-0.83)
**Total**	608686103	1098349	180.4 (180.1-180.8)	184.0 (183.6-184.3)	

*Incidence rates were age adjusted to the 2000 US population.

**Figure 1:**
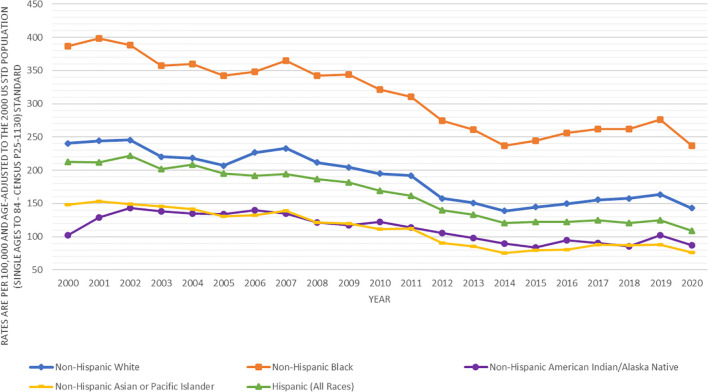
Trends in age-adjusted incidence rates (number of prostate cancer cases per 100,000 population) in men by race and ethnicity in SEER areas from 2000 to 2020.

**Figure 2:**
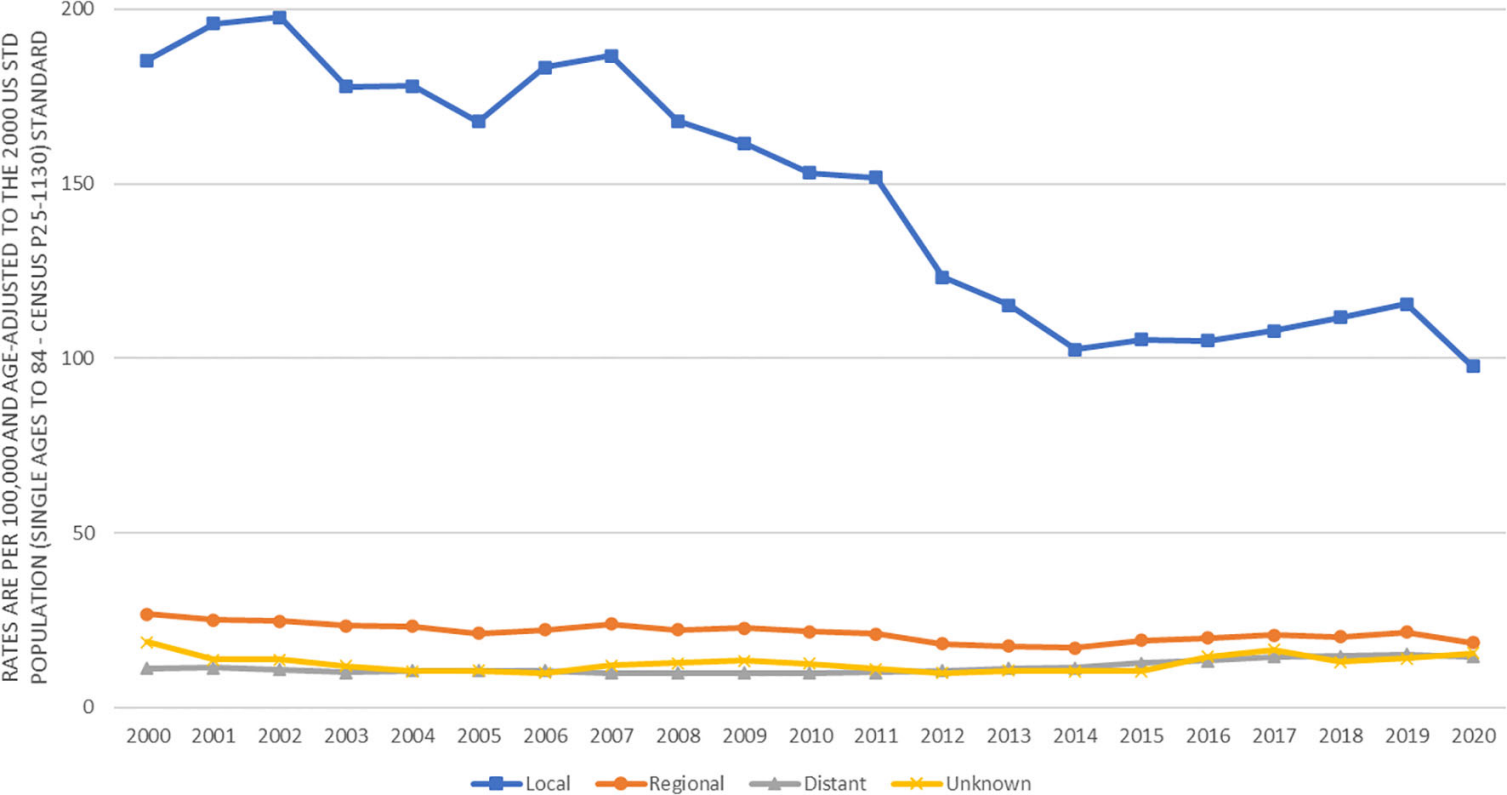
Trends in age-adjusted incidence rates (number of prostate cancer cases per 100,000 population) in men by cancer stage in SEER areas from 2000 to 2020.

**Figure 1** presents the prostate cancer incidence trend from 2000 to 2020 by race and ethnicity. The age-adjusted prostate cancer incidence rates dramatically decreased from 2000 to 2013 for all racial and ethnic groups of men. However, the age-adjusted prostate cancer incidence rates increased from 2014 to 2019, in which the increasing incidence trend looked sharper in black and white men, flatter in Asians, and leveled in AIAN and Hispanics. This figure did show that prostate cancer incidence rates dropped from 2019 to 2020 for all stages (local, regional or distant) except for unknown stage, which was likely due to COVID-19 pandemic lockdown or social distancing, leading to the underreported cancer detection or diagnosis. In addition, the decreasing age-adjusted incidence trend from 2000 to 2013 was much deeper for local stage prostate cancer than for higher stage prostate cancer (**Figure 2**). From 2014 to 2020, age-adjusted incidence rate appeared to increase for all stage prostate cancer, but an increasing incidence rate trend for distant stage prostate cancer was slightly higher than for regional stage prostate cancer (**Figure 2**).

**Table 2** presents the prostate cancer incidence rates and annual percentage change by race and ethnicity and 2 time periods based on what was shown in **Figure 1**. In the decreasing period from 2000 to 2013 for prostate cancer incidence, the decreasing annual percentage change was statistically significant for all racial and ethnic groups of men. In the increasing period from 2014 to 2020 for prostate cancer incidence, the increasing annual percentage change for white, black AIAN, and Asian men and a decreasing annual percentage change for Hispanic men were not statistically significant. However, in the increasing period from 2014 to 2019, the increasing annual percentage change for prostate cancer incidence was statistically significant in white, black and Asian men, but was not statistically significant for AIAN and Hispanics (data not shown). In both time periods (2000-2013 and 2014-2020), the age-adjusted incidence rate of prostate cancer was the highest in blacks, followed by whites, Hispanics, AIAN, and Asians.

**Table 2:** Annual percentage change (APC) in age-adjusted incidence rates* (number of prostate cancer cases per 100,000 population) in men by time period, race and ethnicity.

Race and Ethnicity	2000-2013					2014-2020				
	N. Population	N. Cases	Incidence rate (95% CI)	APC (95% CI)	P value	N. Population	N. Cases	Incidence rate (95% CI)	APC (95% CI)	P value
**Non-Hispanic White**	234264852	535857	208.6 (208.0-209.2)	-3.0 (-4.0, -1.9)	<0.05	120373952	242580	150.7 (150.1-151.3)	1.4 (-1.4, 4.3)	0.30
**Non-Hispanic Black**	38733545	99081	338.1 (335.9-340.4)	-2.6 (-3.4, -1.8)	<0.05	23167816	56030	253.8 (251.6-256.0)	0.9 (-2.1, 4.0)	0.50
**Non-Hispanic American Indian/Alaska Native**	3120302	2688	122.6 (117.6-127.8)	-1.8 (-3.2, -0.3)	<0.05	1752892	1512	90.6 (85.8-95.5)	0.9 (-2.7, 4.6)	0.60
**Non-Hispanic Asian/Pacific Islander**	36557221	35597	123.6 (122.3-124.9)	-4.0 (-5.0, -2.9)	<0.05	24285525	19670	82.2 (81.1-83.4)	1.1 (-2.5, 4.8)	0.50
**Hispanic (All Races)**	77231261	66698	181.0 (179.6-182.5)	-3.4 (-4.3, -2.5)	<0.05	49198737	38636	120.2 (119.0-121.5)	-1.0 (-3.4, 1.4)	0.30
**Total**	389907181	739921	207.7 (207.3-208.2)	-3.1 (-4.0, -2.1)	<0.05	218778922	358428	148.2 (147.7-148.7)	1.0 (-1.8, 3.8)	0.40

*Incidence rates were age adjusted to the 2000 US population.

**Table 3** presents the age-adjusted incidence rates by race and ethnicity that was stratified by 3 time periods and 3 age groups. In all 3 time periods (2000-2006, 2007-2013, and 2014-2020) and in all age groups regardless of young (20-44 years), middle age (45-54), or older age (≥55 years), prostate cancer incidence rate was significantly higher in blacks, but significantly lower in Hispanics, AIAN, and Asians as compared to whites. For instance, in men aged ≥55 years in 2000-2006, the incidence rate ratio of prostate cancer was significantly higher in blacks (1.57, 95% CI: 1.55-1.59), but significantly lower in Hispanics (0.92, 0.91-0.93), AIAN (0.59, 0.55-0.62), and Asians (0.65, 0.64-0.66) as compared to whites. Similarly, in men aged ≥55 years in 2014-2020, the incidence rate ratio of prostate cancer was 1.61 (95% CI: 1.60-1.63) for blacks, 0.82 (0.81-0.83) for Hispanics, 0.61 (0.57-0.64) for AIAN, and 0.56 (0.56-0.57) for Asians as compared to whites.

**Table 3:** Age-adjusted incidence rates (number of prostate cancer cases per 100,000 men) by age groups, race and ethnicity, and time period.

Race and Ethnicity	Age 20-44 yrs				Age 45-54 yrs				Age ≥55 yrs			
	N. Population	N. Cases	Incidence rate (95% CI)	Rate ratio (95% CI)	N. Population	N. Cases	Incidence rate (95% CI)	Rate ratio (95% CI)	N. Population	N. Cases	Incidence rate (95% CI)	Rate ratio (95% CI)
2000-2006												
**Non-Hispanic White**	54767957	1214	2.1 (2.0-2.2)	1.00 (REF)	24496765	21106	84.1 (82.9-85.2)	1.00 (REF)	36609864	248503	706.6 (703.8-709.4)	1.00 (REF)
**Non-Hispanic Black**	10849510	567	5.7 (5.2-6.2)	2.74 (2.48-3.03)	3578510	6052	172.5 (168.1-176.9)	2.05 (1.99-2.11)	3804222	39478	1108.8 (1097.3-1120.4)	1.57 (1.55-1.59)
**Non-Hispanic American Indian/Alaska Native**	879670	9	1.1 (0.5-2.1)	0.54 (0.25-1.02)	305013	121	39.8 (33.0-47.6)	0.47 (0.39-0.57)	317263	1113	413.4 (387.4-440.6)	0.59 (0.55-0.62)
**Non-Hispanic Asian/Pacific Islander**	9457608	52	0.6 (0.5-0.8)	0.31 (0.23-0.40)	3124013	878	28.0 (26.2-30.0)	0.33 (0.31-0.36)	3872249	15922	456.6 (449.3-463.9)	0.65 (0.64-0.66)
**Hispanic (All Races)**	24721006	220	1.3 (1.1-1.4)	0.61 (0.53-0.70)	5234947	2614	52.7 (50.7-54.7)	0.63 (0.60-0.65)	4836639	27505	650.1 (642.0-659.2)	0.92 (0.91-0.93)
**Sub-total (2000-2006)**	100675751	2062	2.2 (2.1-2.3)		36739248	30771	83.2 (82.3-84.1)		49440237	332521	708.6 (706.2-711.0)	
2007-2013												
**Non-Hispanic White**	50468301	1153	2.4 (2.2-2.5)	1.00 (REF)	24620238	22273	84.8 (83.7-85.9)	1.00(REF)	43301727	241608	579.5 (577.2-581.9)	1.00 (REF)
**Non-Hispanic Black**	11169996	610	6.3 (5.8-6.9)	2.68 (2.43-2.96)	4268472	7921	181.2 (177.2-185.2)	2.14 (2.08-2.19)	5062835	44453	923.8 (914.6-933.0)	1.59 (1.58-1.61)
**Non-Hispanic American Indian/Alaska Native**	865478	10	1.4 (0.7-2.5)	0.59 (0.28-1.08)	334304	132	38.2 (31.9-45.3)	0.45 (0.38-0.53)	418574	1303	358.6 (337.8-380.4)	0.62 (0.58-0.66)
**Non-Hispanic Asian/Pacific Islander**	10755611	54	0.6 (0.4-0.7)	0.24 (0.18-0.32)	3806434	1168	29.7 (28.0-31.4)	0.35 (0.33-0.37)	5541306	17523	347.1 (341.8-352.4)	0.60 (0.59-0.61)
**Hispanic (All Races)**	27850173	296	1.4 (1.2-1.5)	0.58 (0.51-0.66)	7377640	3788	52.7 (51.1-54.4)	0.62 (0.60-0.64)	7210856	32275	513.0 (507.1-518.9)	0.89 (0.87-0.90)
**Sub-total (2007-2012)**	101109559	2123	2.4 (2.3-2.5)		40407088	35282	83.8 (83.0-94.7)		61535298	337162	576.2 (574.2-578.2)	
2014-2020												
**Non-Hispanic White**	49494674	553	1.3 (1.2-1.4)	1.00 (REF)	21148611	14126	60.8 (59.8-61.8)	1.00 (REF)	49730667	227901	462.4 (460.4-464.3)	1.00 (REF)
**Non-Hispanic Black**	12342497	383	4.2 (3.7-4.6)	3.16 (2.77-3.61)	4171599	6628	150.1 (146.5-153.8)	2.47 (2.40-2.54)	6653720	49019	745.3 (738.3-752.4)	1.61 (1.60-1.63)
**Non-Hispanic American Indian/Alaska Native**	911392	3	0.5 (0.1-1.3)	0.35 (0.07-0.97)	303127	114	34.3 (28.3-41.4)	0.57 (0.46-0.68)	538373	1395	279.8 (264.4-295.9)	0.61 (0.57-0.64)
**Non-Hispanic Asian/Pacific Islander**	12494563	38	0.4 (0.3-0.5)	0.27 (0.19-0.37)	4291415	914	20.6 (19.3-22.0)	0.34 (0.32-0.36)	7499547	18718	260.8 (257.0-264.7)	0.56 (0.56-0.57)
**Hispanic (All Races)**	29853694	171	0.7 (0.6-0.8)	0.56 (0.47-0.66)	8881594	3268	36.5 (35.3-37.8)	0.60 (0.58-0.62)	10463449	35197	377.1 (372.9-381.3)	0.82 (0.81-0.83)
**Sub-total (2013-2019)**	105096820	1148	1.3 (1.3-1.4)		38796346	25050	60.6 (59.8-61.3)		74885756	332230	454.3 (452.7-455.9)	
**Total (2000-2020)**	306882130	5333	2.0 (1.9-2.0)		115942682	91103	75.7 (75.2-76.2)		185861291	1001913	563.0 (561.9-564.1)	

*Incidence rates were age adjusted to the 2000 US population in each of the three age groups.

**Table 4** presents the age-adjusted prostate cancer incidence rates by race and ethnicity, which were stratified by tumor stages and time periods. Among men with lower tumor stages (local or regional stages) across 3 different time periods, prostate cancer incidence rate was significantly higher in blacks, but significantly lower in Hispanics, AIAN, and Asians as compared to whites. For example, among men with regional stage prostate cancer in 2014-2020, the incidence rate ratio of regional stage prostate cancer was significantly higher in blacks (1.22, 95% CI: 1.19-1.26), but significantly lower in Hispanics (0.73, 0.71-0.76), AIAN (0.56, 0.49-0.65), and Asians (0.59, 0.57-0.61) as compared to whites. However, among men with higher (distant) stage prostate cancer, not just blacks but Hispanic men had significantly higher incidence rate of distant stage prostate cancer than whites in 2000-2006 and 2007-2013, while AIANs did not have significant different incidence and Asians had significantly lower distant prostate cancer incidence in 3 time periods. For instance, among men in 2007-2013, the risk of distant stage prostate cancer was statistically significantly elevated in blacks (2.22, 2.06-2.38) and Hispanics (1.16, 1.06-1.25), not significantly different in AIAN (1.30, 0.92-1.76), but still significantly lower in Asians (0.73, 0.66-0.82) as compared to whites. By comparing the incidence rates of distant stage prostate cancer in 2014-2020 with those in 2007-1013 by race and ethnicity, the incidence ratio was 1.42 times higher in whites, 1.28 times higher in Asians, 1.22 time higher in Hispanics, 1.21 times higher in blacks, and 1.13 times higher in AIAN (**Table 4**).

**Table 4:** Age-adjusted incidence rates (number of prostate cancer cases per 100,000 population) in men by tumor stage, race and ethnicity, and time period.

Race and Ethnicity	Local stage Prostate cancer			Regional Stage Prostate cancer			Distant Stage Prostate cancer		
	N. Cases	Incidence rate (95% CI)	Rate ratio (95% CI)	N. Cases	Incidence rate (95% CI)	Rate ratio (95% CI)	N. Cases	Incidence rate (95% CI)	Rate ratio (95% CI)
2000-2006									
**Non-Hispanic White**	217730	183.5 (182.7-184.3)	1.00 (REF)	30355	24.2 (24.0-24.5)	1.00 (REF)	10,487	9.4 (9.1-9.7)	1.00 (REF)
**Non-Hispanic Black**	36104	283.7 (280.7-286.8)	1.55 (1.53-1.56)	4734	32.4 (31.5-33.4)	1.34 (1.30-1.38)	2,733	25.1 (23.4-26.8)	2.66 (2.47-2.86)
**Non-Hispanic American Indian/Alaska Native**	908	94.3 (87.7-101.2)	0.51 (0.48-0.55)	163	13.5 (11.5-15.9)	0.56 (0.47-0.66)	82	10.3 (6.7-15.0)	1.09 (0.71-1.59)
**Non-Hispanic Asian/Pacific Islander**	13433	113.4 (111.4-115.3)	0.62 (0.61-0.63)	1806	13.7 (13.0-14.3)	0.56 (0.54-0.59)	853	7.8 (7.0-8.8)	0.83 (0.74-0.94)
**Hispanic (All Races)**	23220	156.2 (154.1-158.4)	0.85 (0.84-0.86)	3813	21.8 (21.1-22.6)	0.90 (0.87-0.93)	1,595	12.2 (11.2-13.3)	1.30 (1.18-1.42)
**Sub-total (2000-2006)**	291395	182.3 (181.6-183.0)		40871	23.7 (23.5-23.9)		15,781	10.7 (10.4-11.0)	
2007-2013									
**Non-Hispanic White**	208910	150.2 (149.5-150.8)	1.00 (REF)	32430	21.9 (21.7-22.2)	1.00 (REF)	11,941	9.3 (9.1-9.6)	1.00 (REF)
**Non-Hispanic Black**	42353	246.8 (244.3-249.3)	1.64 (1.63-1.66)	5033	25.7 (25.0-26.5)	1.17 (1.14-1.21)	3,006	20.7 (19.4-22.1)	2.22 (2.06-2.38)
**Non-Hispanic American Indian/Alaska Native**	1057	81.4 (76.2-86.8)	0.54 (0.51-0.58)	165	11.7 (9.8-13.7)	0.53 (0.45-0.63)	129	12.1 (8.6-16.4)	1.30 (0.92-1.76)
**Non-Hispanic Asian/Pacific Islander**	13949	81.2 (79.9-82.6)	0.54 (0.53-0.55)	2481	13.3 (12.7-13.8)	0.61 (0.58-0.63)	1,062	6.9 (6.2-7.6)	0.73 (0.66-0.82)
**Hispanic (All Races)**	27020	120.3 (118.8-121.9)	0.80 (0.79-0.81)	4361	17.1 (16.6-17.6)	0.78 (0.75-0.81)	2,131	10.8 (10.0-11.6)	1.16 (1.06-1.25)
**Sub-total (2007-2013)**	293289	147.9 (147.3-148.4)		44470	20.8 (20.6-21.0)		18,319	10.2 (10.0-10.5)	
2014-2020									
**Non-Hispanic White**	173242	106.7 (106.2-107.2)	1.00 (REF)	34677	20.7 (20.5-20.9)	1.00 (REF)	10,181	13.2 (12.9-13.6)	1.00 (REF)
**Non-Hispanic Black**	41165	182.0 (180.1-183.8)	1.71 (1.69-1.72)	6122	25.3 (24.7-26.0)	1.22 (1.19-1.26)	2,445	25.1 (23.9-26.4)	1.90 (1.79-2.01)
**Non-Hispanic American Indian/Alaska Native**	998	58.1 (54.4-62.0)	0.54 (0.51-0.58)	214	11.6 (10.1-13.4)	0.56 (0.49-0.65)	117	13.7 (10.5-17.4)	1.03 (0.80-1.31)
**Non-Hispanic Asian/Pacific Islander**	13306	55.1 (54.2-56.1)	0.52 (0.51-0.53)	3077	12.2 (11.8-12.7)	0.59 (0.57-0.61)	997	8.8 (8.1-9.5)	0.66 (0.61-0.72)
**Hispanic (All Races)**	25145	76.3 (75.3-77.3)	0.71 (0.71-0.73)	5449	15.2 (14.7-15.6)	0.73 (0.71-0.76)	1,808	13.2 (12.4-14)	1.00 (0.94-1.06)
**Sub-total (2014-2020)**	253856	103.8 (103.4-104.2)		49539	19.5 (19.3-19.6)		15,603	13.9 (13.6-14.1)	
**Total (2000-2020)**	838540	139.7 (139.4-140.0)		134880	21.1 (21.0-21.2)		49,703	10.5 (10.2-10.7)	

*Incidence rates were age adjusted to the 2000 US population.

Among those with unknown stage prostate cancer, the incidence rates by race and ethnicity had similar patterns to those of distant stage prostate cancer. For example, among men in 2007-2013, the risk of unknown stage prostate cancer was significantly higher in blacks (2.23, 2.06-2.40) and Hispanics (1.70, 1.58-1.83), not significantly different in AIAN (1.06, 0.71-1.51), but significantly lower in Asians (0.90, 0.82-1.00) as compared to whites. In addition, both **Figures 1** and **2** showed a drop of prostate cancer incidence from 2019 to 2020 likely due to the impact of COVID-19 pandemic lockdown on the access to medical care in 2020. There were some differences in the decreasing incidence rates by race and ethnicity from 2019 to 2020. For example, overall age-adjusted prostate cancer incidence rate decreased by 40.4 cases per 100,000 population from 277.4 in 2019 to 237.0 in 2020 for blacks, 20.9 from 164.2 to 143.3 for whites, 16.8 from 124.8 to 108.0 for Hispanics, 14.9 from 101.7 to 86.8 for AIAN, and 12.6 from 88.4 to 75.8 for Asians.

## Discussion

This study examined the most recent prostate cancer incidence trends among men in SEER areas of the United States from 2000 to 2020 by time periods, race and ethnicity, age groups and tumor stages, and explored racial disparities in the incidence of developing prostate cancer. In the decreasing period from 2000 to 2013 for prostate cancer incidence, the decreasing annual percentage change was statistically significant for all racial and ethnic groups of men. In the increasing period from 2014 to 2020 for prostate cancer incidence, the increasing annual percentage change was statistically significant in white, black and Asian men, but was not statistically significant for AIAN and Hispanics. The increasing annual percentage change for prostate cancer incidence was statistically significant in white, black and Asian men, but was not statistically significant for AIAN and Hispanics in the increasing period from 2014 to 2019. However, if counting the substantial drop of cancer cases in 2020 due to COVID-19 pandemic infection, there was no significant annual percentage change in prostate cancer incidence for all racial and ethnic men from 2014 to 2020. In both time periods (2000-2013 and 2014-2020), age-adjusted incidence rate of prostate cancer was the highest in blacks, followed by whites, Hispanics, AIAN, and Asians, regardless of age groups, tumor stages, and time periods. There were some racial/ethnic differences in the prostate cancer incidence drop from 2019 to 2020 due to the impact of COVID-19 pandemic lockdown on the access to medical care in 2020.

Although a number of studies examined the global and national incidence trend of prostate cancer in men over the past 2 or 3 decades, two major issues are still not clearly understood without in-depth exploration. The first issue is the observed differences in the incidence trend of prostate cancer incidence between the United States and other countries. In a report that combined cancer incidence data from many countries, the age-standardized incidence rate of prostate cancer increased from 1990 (30.5 cases per 100,000 population) to 2017 (37.9 cases per 100,000 population), including high-income countries such as Canada (2, 3). However, in the United States, the age-adjusted incidence rate of prostate cancer increased sharply from 1975 to 1991, decreased tremendously from 1991 to 2013, and increased again from 2014 to 2019 (1). While the increasing trend in prostate cancer incidence in the early 1990s was most likely due to widespread use of prostate-specific antigen screening that led to case surge in detecting men with asymptomatic prostate cancer, the decreasing trend in 1991-2013 was most likely due to the reduced number of early (local) stage prostate cancer through PSA screening (1). However, the incidence of prostate cancer increased from 2014 to 2019, which was most likely due to the screening guideline in which the United States Preventive Services Task Force (USPSTF) recommended against screening for men aged 75 years and older in 2008 and for all men in 2012 (13–17). The drop in 2020 for the incidence of prostate cancer was observed in the most recent data, which was most likely due to the impact of COVID-19 pandemic lockdown or restriction to medical facilities that substantially reduced the number of people going for regular cancer screening or medical visits for suspicious symptoms related to prostate diseases (23–26). Indeed, we observed the incidence drops from 2019 to 2020 by race and ethnicity with the highest drop in blacks, followed by whites, Hispanics, AIAN, and Asians. Although our study cannot directly address why there were racial differences in the decreased incidence during the COVID-19 pandemic season, other studies demonstrated that the pandemics disproportionally affected blacks who had larger declines in prostate cancer screening and biopsies than other ethnic populations (25, 26). The continued effects of COVID-19 infection and incidence trend after it will need to be monitored in the next few years.

The second major issue is how much prostate cancer incidence trends differed by race and ethnicity. Disparities in cancer incidence have been observed over the past few decades for prostate cancer and many other cancers (18–22, 24–28). Numerous studies have consistently found substantial racial disparities in prostate cancer incidence with a higher rate in blacks and a lower rate in Asians and AIANs (1–3, 18, 19, 27–32). Our study also found significant differences in prostate cancer incidence by race and ethnicity in the most recent data from SEER areas in the United States. We additionally found that these racial disparities differed by tumor stage and time periods. Specifically, among men with local or regional stages across all time periods, prostate cancer incidence rate was significantly higher in blacks, but significantly lower in Hispanics, AIAN, and Asians as compared to whites. However, among men with distant stage prostate cancer, both blacks and Hispanic men were significantly more likely to develop distant stage prostate cancer than whites, while AIANs did not have a significant different incidence in all 3 time periods (2000-2006, 2007-2013 and 2014-2020), but Asians were still significantly less likely to develop distant stage prostate cancer. A previous study by Kratzer and colleagues specifically compared the prostate cancer incidence rates in 2014-2018 between AIAN and white men, and found a significantly lower overall age-adjusted prostate incidence rate in AIAN (89.1 per 100,000 population) than in whites (104.9 per 100,000 population) with a rate ratio of 0.85 (P<0.05) (32). This study also presented some unique and interesting findings on prostate cancer incidence rates between AIAN and white men by geographic areas. For example, they found that AIAN men had significantly lower prostate cancer incidence rates than whites in Alaska, East, Pacific Coast, and Southwest regions, but had significantly higher prostate cancer incidence rates than whites in Northern and Southern Plains (32). These persistent racial disparities in prostate cancer incidence rates have been attributed to differences in genetics, environmental hazards or exposures, access to health care, screening behavior and acceptance, and population health knowledge (18, 19, 27–32). There are still challenges for the research community to more clearly determine the factors responsible for racial disparities and to reduce or eliminate racial disparities in cancer incidence rates.

There are some limitations to be noted in this study. First, ethnic differences in cancer incidence among the subgroups of Hispanic men such as Mexican or Cuban Americans, or among the subgroups of Asian/Pacific Islanders such as Chinese, Korean, Japanese or Pilipino Americans have been reported (2, 20–22, 31), but these subgroup differences in prostate cancer incidence cannot be addressed because of no information on population denominators in SEER*Stats data. Second, place of birth and time of migration were reported to affect the cancer incidence rates by race and ethnicity (33–35), but because of lack of data for population denominators and prostate cancer cases by place of birth and time of migration, our study cannot address the prostate cancer incidence disparities by U.S.-born or foreign-born men or by the timing of migration for those men who immigrated from other countries. Third, prostate cancer cases dropped substantially in the year of 2020. This decrease in incident cancer cases might not be a true phenomenon, but rather a result of underreporting because COVID-19 pandemic lockdown or social distancing requirements significantly affected the access to medical facilities for cancer screening and treatment (23–26). Hence, the incidence rate drop from 2019 to 2020 should be interpreted with great caution. Finally, because the SEER data do not have information or variables on genetic factors, environmental exposures, health insurance, access to health care, screening patterns, and people’s health knowledge or acceptance, the effects of these factors on racial disparities in prostate cancer incidence cannot be addressed.

In conclusion, the decreasing annual percentage change for prostate cancer incidence from 2000 to 2013 was statistically significant for all racial and ethnic groups of men. In the increasing period from 2014 to 2020 for prostate cancer incidence, the increasing annual percentage change was statistically significant in white, black and Asian men, but was not statistically significant for AIAN and Hispanics. The increasing annual percentage change for prostate cancer incidence was statistically significant in white, black and Asian men, but was not statistically significant for AIAN and Hispanics in the increasing period from 2014 to 2019. The largest incidence drop from 2019 to 2020 was observed in blacks, followed by whites, Hispanics, AIAN, and Asians. Age-adjusted incidence rate of prostate cancer was the highest in blacks, followed by whites, Hispanics, AIAN, and Asians, regardless of age groups, tumor stages, and time periods. There will be more challenges ahead for the research community to identify specific risk factors related to race and ethnicity in order to decrease or even eliminate racial disparities for cancer burden. There will also be a need to monitor and investigate the prostate cancer incidence trend during and after COVID-19 pandemic season.

## Data availability statement

The datasets presented in this article are not readily available because this research used the public-user dataset which all researchers can apply for and get access to through the National Cancer Institute for the SEER datasets. Requests to access the datasets should be directed to https://seer.cancer.gov/.

## Ethics statement

Ethical approval was not required for the study involving humans in accordance with the local legislation and institutional requirements. Written informed consent to participate in this study was not required from the participants or the participants’ legal guardians/next of kin in accordance with the national legislation and the institutional requirements.

## Author contributions

XD: Conceptualization, Formal analysis, Funding acquisition, Investigation, Methodology, Project administration, Resources, Software, Supervision, Validation, Visualization, Writing – original draft, Writing – review & editing. DG: Data curation, Formal analysis, Investigation, Methodology, Project administration, Software, Validation, Visualization, Writing – original draft, Writing – review & editing. ZL: Data curation, Formal analysis, Investigation, Methodology, Project administration, Software, Validation, Visualization, Writing – original draft, Writing – review & editing.
